# A Values-Affirmation Intervention Does Not Benefit Negatively Stereotyped Immigrant Students in the Netherlands

**DOI:** 10.3389/fpsyg.2016.00691

**Published:** 2016-05-13

**Authors:** Elisabeth M. de Jong, Francine C. Jellesma, Helma M. Y. Koomen, Peter F. de Jong

**Affiliations:** Research Institute of Child Development and Education, University of AmsterdamAmsterdam, Netherlands

**Keywords:** stereotype threat, values-affirmation, immigrant students, school achievement, problem behavior

## Abstract

Previous research showed that a values-affirmation intervention can help reduce the achievement gap between African American and European American students in the US. In the present study, it was examined if these results would generalize to ethnic minority students in a country outside the US, namely the Netherlands, where there is also an achievement gap between native and ethnic minority students. This type of intervention was tested in two separate studies, the first among first-year pre-vocational students (*n* = 361, 84% ethnic minority), and the second among sixth grade students (*n* = 290, 96% ethnic minority). Most minority participants had a Turkish-Dutch or Moroccan-Dutch immigrant background. In the second study, a third condition was added to the original paradigm, in which students elaborated on either their affirmation- or a control exercise with the help of a teaching assistant. We also examined whether values affirmation affected the level of problem behavior of negatively stereotyped ethnic minority youth. Contrary to what was expected, multilevel analyses revealed that the intervention had no effect on the school achievement or the problem behavior of the ethnic minority students. Possible explanations for these findings, mainly related to contextual and cultural differences between the Netherlands and the US, are discussed.

## Introduction

In many countries around the world, there is a so-called ‘achievement gap’ between native and ethnic minority students. Ethnic minority students underperform in school in comparison to native students (Marks, 2005; Fryer and Levitt, 2006; Pásztor, 2008; Woolf et al., 2013). This gap remains even after controlling for differences in socio-economic status (SES; e.g., Pásztor, 2008; Azzolini et al., 2012; Woolf et al., 2013). It has been proposed that stereotype threat could be an important cause of this achievement gap. Stereotype threat can be defined as the experience of being at risk of confirming others’ negative stereotypes about a group to which one belongs (Steele and Aronson, 1995). For example, in the US, African American students are negatively stereotyped in the academic domain. When these students find themselves in a situation in which the negative stereotype applies, they have been shown to underperform in comparison to students who are not negatively stereotyped in that situation (Steele and Aronson, 1995).

People under stereotype threat are assumed to worry about the personal and social consequences of obtaining poor test grades and, thereby, about confirming the negative group stereotype. These worries, in turn, place on them an extra cognitive and emotional burden that non-stereotyped individuals do not experience. This burden impairs concentration, and therefore, ironically, causes underperformance (Steele and Aronson, 1995; Aronson et al., 2002; Sherman, 2013). Indeed, studies have shown that stereotype threat can increase stress and reduce the working memory capacity that is needed to perform well on tests (see for an overview Schmader et al., 2008). Chronic experience of stereotype threat may lower feelings of belongingness at school (e.g., Cook et al., 2012), and might cause students to disidentify with the school domain. Once disidentified, school achievement is no longer a determinant of self-esteem and, consequently, motivation for school declines (Steele, 1997; Verkuyten and Thijs, 2004; Osborne and Walker, 2006; Owens and Massey, 2011). Because negative stereotypes continue to exist in society, stereotype threat is a chronic stressor for minority students and thereby contributes to the continuation of the achievement gap.

Stereotype threat has been shown to negatively affect the performance of many groups that are negatively stereotyped, including the school performance of Latino American students (Aronson and Salinas, 1997; Rodríguez, 2014), the mathematics performance of women (e.g., Spencer et al., 1999; see also Keller, 2002), the verbal performance of students with a low SES (Croizet and Claire, 1998), and the athletic and the golf performance of white males (Stone et al., 1999). It seems as though any group can be affected, provided that they are negatively stereotyped in a certain domain.

Although stereotype threat has been mostly investigated in the US, several studies have shown that the school performance of ethnic minority students in Europe can also suffer from stereotype threat. For example, Appel (2012) showed that adolescent immigrant students in Austria, including students with a Turkish background, underperformed on a culture fair intelligence test under stereotype threat. Furthermore, in France, young adult ethnic minority students’ with a North-African or Arabic background (e.g., Moroccan) underperformed on a memory test (Berjot et al., 2011) and a test of verbal ability (Chateignier et al., 2009) under conditions of stereotype threat. Similar stereotype threat effects on performance have also been found for Turkish students in Germany (see Appel et al., 2015 for an overview of published and unpublished studies about stereotype threat effects among immigrant students).

To reduce the negative effects of stereotype threat on the school performance of African American middle school students, previous studies have used a values-affirmation intervention (e.g., Cohen et al., 2006b). Several longitudinal studies have shown that this type of intervention can reduce the achievement gap between non-stereotyped and stereotyped individuals (e.g., Cohen et al., 2006b, 2009; Miyake et al., 2010; Sherman et al., 2013; Harackiewicz et al., 2014).

Most research about stereotype threat and values affirmation has taken place in the United States. However, in other countries, there are also achievement gaps between native and negatively stereotyped ethnic minority students. The present study therefore examines whether a values-affirmation intervention can have the same positive effect on the performance of another group of students outside the US about whom similar negative stereotypes are thought to exist (e.g., Verkuyten and Thijs, 2000), namely ethnic minority students in the Netherlands. In addition to an achievement gap, in the Netherlands there are also differences in the levels of problem behavior of native and ethnic minority students (e.g., Stevens et al., 2003), although different informants (e.g., self-reporting, teacher, parent) do not always agree (Stevens and Vollebergh, 2008). As an extension of the original research (Cohen et al., 2006b), we also examine if a values-affirmation intervention has an effect on the level of problem behavior of these negatively stereotyped students.

### Values-Affirmation Intervention

#### Values Affirmation and School Achievement

Values-affirmation interventions are based on self-affirmation theory (Steele, 1988). According to self-affirmation theory, people are motivated to maintain a sense of self-integrity or personal adequacy. When one is negatively stereotyped in a certain domain, such as academic ability, the sense of self-integrity can become threatened. Hence, one can experience stereotype threat in that domain. Values affirmation, which is a form of self-affirmation, helps to re-affirm self-integrity, presumably by giving individuals a broader perspective on the self (Sherman, 2013), and by taking the focus off of the threatened part of the identity (Sherman, 2013; Critcher and Dunning, 2014).

One of the first studies about stereotype threat and values affirmation was conducted by Cohen et al. (2006b). They conducted a large-scale longitudinal intervention study among seventh-graders in the US. In two randomized double-blind field experiments, African American and European American students were randomly assigned at the individual level to either the affirmation or the control condition. During school hours students received either one (Study 1) or two (Study 2) short writing assignments which took only 15 min to complete. In the affirmation condition, students were asked to choose from a list the two or three values that were *most* important to them (e.g., music, athletic abilities, relationships with friends and family) and to write a short paragraph about why those values were personally important to them. In the control condition, participants were asked to choose the two or three values that were *least* important to them and to write about why these values could be important to someone else (Cohen et al., 2006b).

Results showed that the values-affirmation intervention increased the academic performance of the negatively stereotyped African American students and reduced the achievement gap between African American and white students by 40% (i.e., average reduction over the two experiments; Cohen et al., 2006b). Furthermore, of the African Americans in the control condition, 20% scored a grade D or below in the targeted course, whereas in the affirmation condition, this was only the case for nine percent of the students. Results from a follow-up study revealed that the effects were even sustained over a 2-years period, after students had received several extra interventions over the course of the first year (Cohen et al., 2009). As expected, the intervention had no effect on the achievement of European American students.

According to Cohen et al. (2006b), the reason why such a seemingly small and simple intervention could have such a large impact is that the intervention disrupts a negative recursive cycle of stereotype threat and poor performance. They argue that re-affirmation of self-integrity probably causes a small reduction in psychological threat, which leads to a slightly better performance on the subsequent test. This effect can act as a self-fulfilling prophecy, interrupting a downward trend and instigating a more positive cycle. Indeed, African Americans in the control condition showed a significant downward linear trend in performance, whereas in the affirmation condition, this downward linear trend was absent. These results imply that the effects of a small intervention can be far-reaching. Especially when the intervention is planned early in the school year, before any negative recursive cycle can arise and when evaluative stress is assumed to be high, values affirmation has proven to be effective in improving performance (Cook et al., 2012).

After the study of Cohen et al. (2006b), several longitudinal replication studies have been conducted amongst negatively stereotyped groups other than African Americans, using a similar intervention paradigm. For example, Sherman et al. (2013) showed that a values-affirmation intervention had positive effects very similar to those found by (Cohen et al., 2006b, 2009) for Latino students, a group that differs from African American students at least in that most of them have a much more recent migration history (e.g., most of the parents of the students in the described study were first-generation immigrants), and many of them are not completely fluent in English (Sherman et al., 2013). Whereas (Cohen et al., 2006b, 2009) examined one racial group (i.e., African Americans), Sherman et al. (2013) combined Latino and Hispanic students from different countries into one ethnic minority group, arguing that they have a common social identity. Intervention effects were found for the combined group.

The studies described above were carried out at schools with a relatively even distribution of ethnic minority (i.e., African American, Latino) and majority (e.g., white) students. However, a study by Bowen et al. (2013) has shown that the intervention can have similar positive effects at schools where the student body consists mainly of ethnic minority students. To be able to compare their results to those of Cohen et al. (2006b), Bowen et al. (2013) focused their analyses on the African American and ‘white’ group in their sample. As opposed to the above described studies, the ‘white’ group in the study of Bowen et al. (2013) was much smaller, and consisted not only of white students, but also of Asian and mixed race students. Still, they assumed that all students at the examined school could experience some form of negative stereotyping, either because of their ethnic background or their low SES. Therefore, they hypothesized that the intervention would positively affect the performance of all participants. Indeed, this is what they found.

Another study, carried out by Harackiewicz et al. (2014), demonstrated that students with a relatively low SES can also benefit from the intervention. There is often an achievement gap between students who are the first in their family to go to college (usually coming from families with a low SES) and students who are not. Harackiewicz et al. (2014) showed that this gap can also be attenuated by a values-affirmation intervention. The authors describe two reasons why the intervention might have worked for these students. First, as in the previously described studies, first generation students could be suffering from stereotype threat, for example because of negative stereotypes regarding their low SES. Second, they argue that these students could also be experiencing a form of what they call ‘cultural identity threat,’ because of a conflict between their own interdependent motives to go to college and the independent norms of the college. These conflicting motives could lead to a lower sense of ‘fit’ or belonging in the academic context. Values affirmation has been shown to improve feelings of belongingness (e.g., Cook et al., 2012).

Yet another study that produced results very similar to those of Cohen et al. (2006b) was done by Miyake et al. (2010). They examined yet another negatively stereotyped group, namely women in the field of physics and mathematics. A values-affirmation intervention positively affected the performance of women in college physics, whereas this was not true for men. The gender gap was reduced by 61%. An earlier non-longitudinal experimental study had also shown the positive effect of values affirmation on the math performance of female introductory psychology students (Martens et al., 2006).

The studies described above were all performed in the US among middle school students (i.e., grades six to eight) or college students. All of these studies showed that the intervention had a positive effect on the achievement of the negatively stereotyped participants. However, it is unclear to what extent such effects would generalize to countries outside the US with, for example, different ethnic minority groups. One would expect similar results considering the variety of negatively stereotyped groups that have benefited from the intervention in the US. However, not many studies have examined effects outside the US.

One of the few studies is a non-longitudinal study among French female nursing students (Taillandier-Schmitt et al., 2012). For French nurses, mathematical skills are becoming increasingly important, while there is still a negative stereotype about women and mathematics. The researchers manipulated stereotype threat by using test instructions that either explicitly mentioned the negative stereotype or not. Thereafter, some students received a values-affirmation exercise, whereas others received a control exercise. The results showed that a values-affirmation intervention did indeed eliminate the negative effect of stereotype threat on the nurses’ mathematical performance (Taillandier-Schmitt et al., 2012). It thus seems that the values-affirmation intervention can positively affect the performance of negatively stereotyped students outside the US as well. However, a disadvantage of this study was that there was no control group of male nurses included. Therefore, one cannot exclude the possibility that male nurses, who were not expected to suffer from stereotype threat, would have benefitted equally from the intervention.

The only longitudinal study about stereotype threat and values affirmation outside the US that we know of was carried out by Woolf et al. (2013). This study took place among native and ethnic minority medical students at a British medical school. The results were mixed. Woolf et al. (2013) examined the effects of the values-affirmation intervention on a written- and a clinical assessment. Against all expectations, they found that on the written assessment, white students in the affirmation condition performed worse than white students in the control condition, whereas there was no effect for ethnic minority students. On the clinical assessment they found that all students, white and ethnic minority, benefited from the intervention. Although, this study was presented as a direct replication of the studies of (Cohen et al., 2006b, 2009), it differed on at least two important aspects. First, the study was focused on an older population, namely university students. Second, the ethnic minority group that was studied (most had an Asian background) is not very comparable to the earlier described populations (e.g., Cohen et al., 2006b, 2009) in terms of being negatively stereotyped. Woolf et al. (2013) mention that stereotypes about these groups are, for example, that they are hardworking and do a lot of rote-learning. One could hardly call these negative stereotypes and could even consider these to be positive stereotypes. Therefore, one could question the applicability of the intervention in this situation.

In a recent meta-analysis, Appel et al. (2015) showed that stereotype threat effects are also found among immigrant students in Europe, and suggested that a values-affirmation intervention might benefit these immigrant students in the same way as it benefited ethnic minority students in the US. The present study focuses on examining to what extent the intervention effects would generalize to ethnic minority students (i.e., students with an immigrant background) in the Netherlands. Unlike the sample that was studied in the British study (Woolf et al., 2013), in the present study the ethnic minority students are more comparable to the students examined in the US intervention studies with regard to age as well as to negative stereotypes that exist about them in society. Furthermore, because ethnic minority students in the Netherlands are not only negatively stereotyped in the intellectual but also in the behavioral domain (e.g., Verkuyten and Thijs, 2000), we examine intervention effects on performance as well as on problem behavior.

#### Values Affirmation and Problem Behavior

Ethnic minority students are often negatively stereotyped in terms of problematic behavior (e.g., Verkuyten and Thijs, 2000, 2010; Nievers et al., 2010). However, to our knowledge, there are no studies examining the relationship between stereotype threat and problem behavior. For two reasons, we assume that stereotype threat can increase problem behavior and, therefore, that a values-affirmation intervention could also be suitable to reduce the level of problem behavior. First, previous research has shown that negative stereotypes about behavior can be directly related to problem behavior among the stereotyped individuals. Kamans et al. (2009) found that Moroccan-Dutch youth who felt negative about the native Dutch out-group and who felt personally stereotyped by them, were inclined to legitimize behavior in line with the negative stereotypes, such as loitering, aggressive and criminal behavior. When negatively stereotyped by a disliked out-group, desires to protect the in-group identity might thus ironically lead to an increase of problem behavior and a confirmation of the negative group stereotype.

One could argue that the process described above is a form of stereotype threat, because the identity threat stems from negative stereotypes and also leads to confirmation of the negative stereotype. Given that values affirmation is thought to relieve identity threats by making the individual aware of multiple sources of self-integrity (Sherman, 2013; Critcher and Dunning, 2014), one would expect that values affirmation could reduce such feelings of threat caused by negative stereotypes in the behavioral domain. Protecting the in-group identity by behaving negatively to out-group members (e.g., Kamans et al., 2009) would become less relevant, because values affirmation makes one aware that group membership is only one aspect of the self, and that one’s self-integrity does not depend on this specific group membership only.

A second reason to expect that stereotype threat can increase problem behavior is that previous studies have shown that coping with stereotype threat in one domain (e.g., the intellectual domain), can deplete resources for self-control in subsequent (e.g., social) situations, unrelated to the stereotype (Inzlicht and Kang, 2010). Usually, aggressive impulses are regulated by self-control; the capacity that is needed to overcome environmental temptations and urges, emotions and automatic response tendencies (Baumeister and Heatherton, 1996). However, Inzlicht and Kang (2010) demonstrated that when self-control resources have been depleted by stereotype threat, this can reduce inner restraints against aggression.

Schmeichel and Vohs (2009) have shown that when self-control resources have been depleted in one situation, values affirmation can restore self-control resources for subsequent situations. In their first experiment, participants either did a task that depleted self-control resources or a control task. After the task, half of the participants did a values-affirmation exercise and half did a control exercise, comparable with the exercises used by Cohen et al. (2006b). Subsequently, participants did a task in which self-control was needed. The results showed that for the participants who had received the non-depleting first task, there was no difference in self-control on the second task between participants in the self-affirmation and control condition. However, for the participants who had done the depleting first task, self-affirmation had a positive effect on self-control on for the second task.

In the present study, we examine if a values-affirmation intervention can reduce problem behavior of negatively stereotyped ethnic minority students. We assume that when self-control resources are depleted by stereotype threat, value-affirmation could have similar positive effects on behavior as in the study of Schmeichel and Vohs (2009). Although, feelings of threat or the degree of the depletion of resources are not explicitly measured in the present study, we do examine if the values-affirmation intervention affects the level of problem behavior of ethnic minority students in the Netherlands.

### Present Study

Two of the largest ethnic minority groups in the Netherlands are Moroccan and Turkish immigrants who came to Western Europe in the 20th century as guest workers to fill positions in the lower segments of the labor market. They mainly came from the lower socio-economic classes (Organisation for Economic Co-operation and Development [OECD], 2002). Turkish and Moroccan immigrants are negatively stereotyped in the Netherlands, in both the intellectual and the behavioral domain (e.g., Verkuyten and Thijs, 2000; Centraal Bureau Voor de Statistiek [CBS], 2005; Nievers et al., 2010). Moreover, they also experience negative stereotyping related to Muslim extremism (Kamans et al., 2009). Previous studies have shown stereotype threat effects on the performance of immigrant students of the same background (i.e., Moroccan and Turkish) in neighboring countries of the Netherlands (i.e., Germany, France, Austria; Chateignier et al., 2009; Berjot et al., 2011; Appel, 2012; see for an overview Appel et al., 2015), countries of which the cultures are similar to that of the Netherlands.

We expect the intervention to have positive effects on the school performance of ethnic minority students in the Netherlands similar to those seen in the US, because both are negatively stereotyped groups. Ethnic minority students in the Netherlands are probably most comparable to Latino American students in the US both in terms of their recent migration history and their second language fluency. For Latino American students, the intervention has been shown to have positive effects comparable with those found for African American students (Sherman et al., 2013). Moreover, apart from experiencing stereotype threat, ethnic minority students in the Netherlands could also be expected to experience cultural mismatch at school, as described in the study of Harackiewicz et al. (2014), or negative stereotyping because of their relatively low SES (Bowen et al., 2013; Harackiewicz et al., 2014). These are all additional reasons to expect the intervention to have the intended positive effect on these students. By means of two studies, the effects of a values-affirmation intervention on the school achievement and problem behavior of ethnic minority students (i.e., students with an immigrant background) in the Netherlands are examined. The aim of the first study was to examine if the findings of (Cohen et al., 2006b, 2009) would generalize to a sample of secondary school students outside the US with a comparable degree of negative stereotyping.

## Study 1

This study was conducted among first-year pre-vocational students. In the Netherlands there are different levels of secondary education. Students make a transition to secondary school after sixth grade, and their level of performance at the end of primary school determines to which school level they go. Previous studies (e.g., Cohen et al., 2006b, 2009) have shown that poorly performing students benefited most from the values-affirmation intervention. Therefore, the present study was conducted at the lowest level of secondary education (pre-vocational education), directly after the transition to secondary school.

We hypothesized that the values-affirmation intervention would have similar positive effects on the school achievement of ethnic minority students in the Netherlands as in the US. For native students, our expectations were not clearly defined. On the one hand, one would expect native Dutch students not to benefit from the values-affirmation intervention because they are not negatively stereotyped in society on the basis of their ethnic background. However, native students in pre-vocational education often also have a relatively low SES, which could be related to experiencing negative stereotyping in the intellectual domain. If native students also experience a form of stereotype threat because of their low SES and supposedly low IQ, then one would expect the intervention to have a positive effect on this group as well (e.g., Bowen et al., 2013; Harackiewicz et al., 2014). Moreover, because girls are often negatively stereotyped in the science and mathematics domain and a values-affirmation has been shown to improve the performance of female students in science and mathematics (Martens et al., 2006; Miyake et al., 2010), in the present study we also examined if there were intervention effects on girl’s mathematics performance.

Furthermore, the effects of the intervention on two underlying factors of the stereotype threat process, namely school belongingness (Cook et al., 2012) and identification with school (Steele, 1997; Osborne and Walker, 2006; Owens and Massey, 2011) were examined. In line with previous studies, we expected that the values-affirmation intervention would positively affect these two factors for ethnic minority students. Again, our expectations for the native Dutch group were not clearly defined.

We did not expect to find large achievement gaps between native and ethnic minority students at the beginning of the school year within classes, because students had already been subdivided into different school levels on the basis of their primary school performance. However, we assumed that being sent to the lowest level of education probably made students very aware of the fact that there are also students with higher performance levels.

As an extension to the study of Cohen et al. (2006b), we also examined the effects of the values-affirmation intervention on the level of problem behavior of ethnic minority students in the Netherlands. Our hypothesis was that the values-affirmation intervention would decrease the level of problem behavior amongst these students. For native Dutch students, we expected no intervention effects because they are not, or at least less, negatively stereotyped in the behavioral domain. We did not know whether to expect differences in problem behavior between native Dutch and ethnic minority students at the beginning of the school year. Although, previous studies have shown that teachers report more problem behavior for ethnic minority students than for native students (Stevens et al., 2003), we assumed that the level of problem behavior amongst native Dutch students at the lowest level of education might also be relatively high.

### Materials and Methods

#### Participants

**FIGURE 1:**
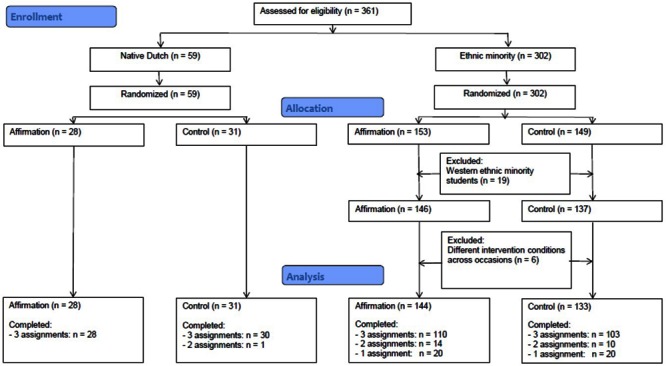
**Flowchart of the random assignment procedure of Study 1**.

In total, 361 first year pre-vocational students (42.1% boys) participated in the present study (59 native Dutch and 302 ethnic minority). Ages ranged from 11.75 to 15.08 (*M*_age_ = 12.90 years, *SD* = 0.58). Students were assigned to the different conditions on the basis of a randomized controlled trial. Pairs of participants, matched on gender and ethnic background, were formed within classes. Of each pair, one participant was randomly assigned to the values-affirmation and the other to the control condition. Students for whom a match within the classroom was impossible because of an uneven number of students, were matched with students from another class. The assignment procedure is displayed in **Figure 1**.

The study took place in 17 classes from six pre-vocational secondary schools in the Netherlands. Schools were recruited in or near large cities in the western part of the Netherlands (Amsterdam, Rotterdam, The Hague), where percentages of ethnic minority students in secondary schools are high. Because the main focus of this research project was to examine the intervention effects on ethnic minority students, we explicitly recruited schools with a population of at least 45% ethnic minority students. Percentages of non-Western ethnic minority students per participating class ranged from 50 to 100%. With the exception of one school, all participating schools were located in areas with relatively high percentages of non-Western immigrants (between 32 and 74% of the total population of the area, versus 12% nationwide; CBS Statline, 2011). One school was not situated in an area with a high percentage of ethnic minority residents. However, this school was in a city that was easily accessible by bus from areas with high percentages of ethnic minority residents. Many students traveled from these areas to this school.

The ethnic background of the participants was determined by using the background information students provided during the first measurement occasion (e.g., country of birth, country of birth of parents and origin of the greater family). There were 19 students (6.3% of the ethnic minority students) with either incomplete background information or a Western ethnic minority background (e.g., Australia or France). It was decided to exclude these students from the analyses, because it was unclear if negative stereotypes existed about these groups. This left 342 students in the analyses, namely 59 native Dutch students (28 affirmation, 31 control) and 283 students with a non-Western ethnic minority background (146 affirmation, 137 control).

Of the 283 ethnic minority participants, 38.2% had a Moroccan background, 31.8% had a Turkish background and 12.0% had a Surinamese or Antillean background. These three groups were the largest ethnic minority groups in the sample, which corresponds to the largest ethnic minority groups in Dutch society. The remaining 18% of non-Western ethnic minority students in the sample had various ethnic backgrounds (e.g., Pakistan, Iraq, Indonesia, Somalia).

The ethnic minority students were mainly second generation immigrants, meaning that they were born in the Netherlands but had at least one parent born in a non-Western country. Thirty-two (11%) of the ethnic minority students indicated that they had been born in the country of origin of the parents. However, these students resided in the Netherlands for on average 10.08 years (*SD* = 2.19). All other students were born in the Netherlands. No differences were found in the outcome measures between the group of students that was born in a non-Western country and the group that was born in the Netherlands. Therefore, the groups were combined.

#### Negative Stereotypes in the Netherlands

To examine if ethnic minority students in the Netherlands indeed experienced more negative stereotyping than native Dutch students, a study was conducted among another sample of seventh grade students (*N* = 283). Of these students, 49.8% had a native Dutch background, 19.8% had a Moroccan-Dutch background and 30.4% had a Turkish-Dutch background. Participants were asked to indicate if they did or did not experience the following negative stereotypes about their cultural group: dumb, unreliable, inhospitable, lazy, aggressive, greedy, not hard working, criminal, unable to understand things quickly. Two of the negative stereotypes (inhospitable and greedy) are negative stereotypes that were anticipated to exist about the Dutch. The other stereotypes were expected to exist about ethnic minority students in the Netherlands. Chi-square tests revealed that ethnic minority students thought significantly more often than native Dutch students that others saw their cultural group as dumb, untrustworthy, lazy, aggressive and criminal. On the other hand, native Dutch students more often thought that others saw their group as greedy than ethnic minority students. These results thus show that ethnic minority first-year pre-vocational students in the Netherlands experience negative stereotyping in the intellectual as well as the behavioral domain, and they experience negative stereotyping more often than native Dutch students.

#### Intervention

For the implementation of the intervention, we closely followed the procedures of (Cohen et al., 2006b, 2009; see also Sherman et al., 2013). The intervention consisted of three short writing assignments, spread out over a period of one school year. The writing assignments were adopted from (Cohen et al., 2006b, 2009) and translated into Dutch with the aid of a certified translator. Students in the values-affirmation condition received a list of 12 values (e.g., music, athletic ability, relationships with friends and family) and were asked to choose the two or three that were most important to them. Subsequently, they were asked to write a short paragraph about why these values were important to them. Students in the control condition received the same list of values, but were asked to select the two or three values that were least important to them. Their assignment was to write a short paragraph about why these values that were least important to them could be important to someone else. As a reinforcement of the manipulation, students were asked to indicate on a six-point scale how much they agreed with propositions concerning the chosen values. An example proposition in the affirmation condition was: ‘I care about these values.’ An example in the control condition was: ‘Some people care about these values.’

Following the procedures of Cohen et al. (2006a; supporting online material), teachers were instructed to schedule the interventions directly before a school test. When this was not possible, the researchers either provided a standardized arithmetic test that teachers could give to their students directly before the intervention, or they instructed the teacher to have a short conversation with their students directly before the intervention took place. The topic of this conversation was school testing that would take place in the near future, and was also expected to raise evaluative stress. With the exception of two, all classes received the intervention at least one time before a school test or arithmetic test or after a conversation about school testing.

The first intervention round was timed as early in the school year as possible, because evaluative stress was expected to be high soon after the transition to secondary school and feelings of belongingness are still uncertain during such a transition. Furthermore, previous studies have shown that interventions timed early in the school year are more effective, because at that time they can still prevent the development of negative recursive cycles (Walton and Cohen, 2007; Cook et al., 2012).

Intervention assignments were administered by the teacher and were presented as a school task in order to prevent students from knowing that the interventions were part of a research project. To avoid expectancy effects, teachers were blind to the purpose of the writing assignments and were also unaware of the existence of different conditions. One or two weeks before the assignments were administered, teachers received information on how to administer the assignments, so that they could prepare. They received a document with standard instructions to introduce the assignments and were provided with standard answers to any potential questions that could be posed by students.

Most students received the assignments during Dutch class, since these were Dutch writing assignments. At one school (four classes), this was not possible and therefore students received the assignments during mentor supervision hours. Students received the assignments in white envelopes with their names on them. They were guided through the assignments via written instructions and completed the assignments independently. Students were instructed not to worry about spelling or grammar. The assignments took approximately 15 min to complete. After finishing, the participants were free to seal their envelopes. Teachers collected the assignments and resumed their lesson plans. The assignments were collected from the teachers by a member of the research team without the students knowing.

#### Procedure

**FIGURE 2:**
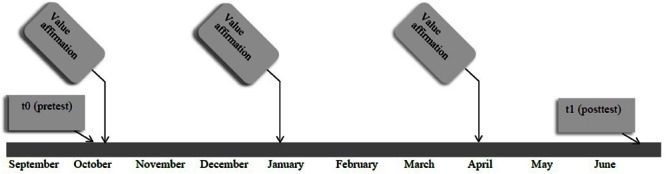
**Time schedule of the intervention Study 1**.

Students received three interventions throughout the school year. Furthermore, belongingness, identification with school and problem behavior were measured by means of questionnaires before the first and after the third intervention (see **Figure 2**). The questionnaires were administered by a member of the research team, who gave a short introduction and was available for student questions. The teacher was also present. The questionnaires took approximately 40 min to complete. Pre- and post-intervention school grades were obtained from the schools. The interventions were presented as a school task and were administered by the teachers. After the last questionnaire, participants received a small gift as a reward.

#### Ethics Statement

Before the onset of the study, the students’ parents were informed about the project by letter and were asked to indicate whether they had any objections to the participation of their child (i.e., passive informed consent). One student did not receive parental permission and was given an alternative assignment while the rest of the class filled out the questionnaires. Because the intervention was presented as a school task and to prevent students from suspecting that the intervention was part of the research project, this student received the control writing exercises. No special approval from the department’s Ethics Review Board needed to be requested at the start of the project, because the research was classified as ‘standard’ in our department: there was no reason to assume that the study would potentially have any negative effects on the participants, no physiological or health measures were taken, and the intervention assignments resembled a normal school task. Furthermore, consulting an Ethics Review Board was no standard procedure at our department at the time the study was to be conducted. Schools gave permission for the research project to take place within school hours. Data were analyzed anonymously. The students were requested to fill in their names on their questionnaires solely for the purpose of linking together their questionnaires from the different measurement occasions. The students were assured that their answers would be kept confidential and that no teacher nor school personnel nor others would read their answers, except for the researchers. After the first measurement occasion, all questionnaires were numbered. Names of the participants are kept in a separate file, which only the first author had access to. Identifying information was never used in any analyses.

#### Measures

##### School grades

School grades for the subjects Dutch, English, and mathematics were collected from the schools before the first and after the third intervention. In the Netherlands, students receive grades on a scale from 1 (very poor) to 10 (excellent). For a portion of the students (226 ethnic minority and 32 native Dutch), scores of the Cito test from sixth grade were available. The Cito test is a nationwide independent exam that is taken by all students at the end of sixth grade. It is a general assessment of what students have learned in primary school. The test consists of 290 multiple choice questions, divided over several modules, including language, arithmetic, world orientation (i.e., geography, history, science) and study skills. Students receive a standard score between 501 and 550. This score contributes to the recommendations teachers make with regard to which school level students should go after sixth grade. *t*-tests showed that both ethnic minority students, *t*(226) = -8.64, *p* < 0.001, as well as native Dutch students, *t*(31) = -7.64, *p* < 0.001, scored significantly lower than the national average on this test.

##### Problem behavior

Self-reported problem behavior was measured with items from the Dutch version of the Youth Self Report (YSR; De Groot et al., 1996). The items were selected from the subscales Rule Breaking Behavior and Aggressive Behavior (11 items, mean Cronbach’s α = 0.78 across measurement occasions; example items: ‘I often skip classes at school’ and ‘I am mean to others’). All items were answered on a three-point scale, ranging from ‘not true’ to ‘true.’

##### Belongingness

Feelings of belongingness at school were measured using two subscales of the School Attitude Questionnaire (Schoolvragenlijst; Smits and Vorst, 1990). These subscales were School Enjoyment (eight items, mean Cronbach’s α = 0.73 across measurement occasions) and Perceived Social Acceptance by Classmates (eight items, mean Cronbach’s α = 0.78 across measurement occasions). All items were answered on a three-point scale (i.e., that is not true, I don’t know, that is true).

##### Identification with school

Identification with school was measured using four items about the perceived importance of performing well at school (mean Cronbach’s α = 0.72 across measurement occasions). There was one general item (“How important do you find it to receive good grades at school?”) and three items that asked the same question for specific school subjects (Dutch, English, and mathematics). All items were answered on a three-point scale, ranging from ‘important’ to ‘not important.’

#### Statistical Analyses

As data were gathered in different classes from different schools, they have a nested structure. Pupils nested within the same class are probably more equal to each other than pupils from other classes or from other schools. Therefore, multilevel or random coefficient models (Hox, 2010) were used. Multilevel analyses take into account dependencies among respondents from the same class (e.g., Snijders and Bosker, 2012). Multilevel analyses were conducted using SPSS Version 20.

### Results

#### Data Preparation

Due to administrative errors, six participants were not included in the same intervention condition on all three intervention occasions and were therefore excluded from the analyses, leaving 336 students in the analyses, namely 59 native Dutch and 277 ethnic minority students (see **Figure 1**). Data are included in the Supplementary Materials (see Data Sheet 1). Of these 336 students, 271 students received all three intervention assignments. Due to absence during one or two intervention occasions, 24 students received only two assignments and 40 students received only one assignment (see also **Figure 1**). However, there were no significant differences between the values-affirmation and the control condition in the mean number of interventions completed, *F*(1,334) = 0.002, *p* = 0.97. Therefore, all 336 students remained in the analyses. There were 28 native Dutch students in the affirmation condition and 31 in the control condition. Of the ethnic minority students, there were 144 students in the affirmation condition and 133 in the control condition.

On both measurement occasions, data for school belongingness, school identification, and problem behavior were available. For all 17 classes, post-intervention school grades were also available. For one variable (i.e., pre-intervention school grades) there were some data missing. Because some schools did not provide information on the dates at which their school tests were administered, it was not possible to determine pre-intervention grades for all classes. Pre-intervention school grades were therefore only available for six (Dutch) or seven (English and mathematics) classes of the 17 classes. However, because these grades were missing for whole classes and not at the individual level, and students were randomized within classes, we expected no differences in background characteristics between students for whom grades on both measurement occasions were available and students for whom only post-intervention grades were available. An independent samples *t*-test showed that there were no age differences between the six classes with pre- and post-intervention Dutch grades available (*N* = 123) and the 11 classes (*N* = 208) with only post-intervention Dutch grades, *t*(329) = -0.63, *p* = 0.53. There were also no age differences between the seven classes with pre- and post-intervention English and mathematics grades available (*N* = 143) and the 10 classes (*N* = 188) with only post-intervention grades available, *t*(329) = -1.85, *p* = 0.07. School grades were missing for five students. Because post-intervention school grades were available for all classes, intervention effects on grades were first examined only on these post-intervention grades. Thereafter, analyses were performed in which we controlled for pre-intervention grades.

All predictor variables (i.e., gender, ethnic background, and condition), except pre-intervention grades and behavior, were added as dummy variables in the analyses. Gender was included in all models as a control variable. All outcome variables were standardized before they were entered into the analyses. In this way, the parameter estimates can be interpreted as effect sizes. For example, a coefficient of 0.5 corresponds to an increase of 0.5 *SD*.

For the analyses of the intervention effects, we followed the procedures of Cohen et al. (2006a, supporting online material). The only difference was that we used multilevel analyses to control for dependencies within classes, whereas Cohen et al. (2006b) used two dummy variables to control for the three different teachers in their study. Because our study included 17 different teachers, multilevel analyses were considered to be a more suitable solution.

#### Pre-intervention Differences between Native and Ethnic Minority Students

Before the effects of the intervention were analyzed, it was examined if there were differences in achievement (i.e., an achievement gap), and problem behavior between native Dutch and ethnic minority students. This was examined with multilevel analyses with two levels (students nested within classes) on pre-intervention school grades and problem behavior. Furthermore, it was also examined if there were pre-intervention differences in school belongingness, and identification with school.

#### Pre-intervention Achievement Gap and Differences in Problem Behavior

Multilevel models with two levels (students nested within classes) did not reveal a pre-intervention achievement gap in Dutch grades (β = 0.03, *p* = 0.87) or English grades (β = -0.07, *p* = 0.65), but there was one in mathematics grades (β = 0.59, *p* < 0.01). Native Dutch students had higher pre-intervention mathematics grades than ethnic minority students. There were no differences in pre-intervention self-reported problem behavior between native and ethnic minority students (β = -0.13, *p* = 0.38). As outlined in the introduction, an absence of differences in achievement or problem behavior within the class does not necessarily imply that the intervention cannot affect the achievement or behavior of ethnic minority students in the present study.

#### Pre-intervention Differences in Belongingness and Identification with School

There were no pre-intervention differences between native and ethnic minority students on the belongingness measures School Enjoyment (β = -0.13, *p* = 0.42), and Perceived Social Acceptance (β = -0.20, *p* = 0.18). There were also no pre-intervention differences on Identification with School (β = -0.18, *p* = 0.23).

#### Pre-intervention Differences between Girls and Boys on Mathematics Performance

There were no pre-intervention differences between boys and girls on mathematics performance (β = -0.12, *p* = 0.44).

#### Intervention Effects

##### Grades

Descriptives of post-intervention grades are displayed in **Table 1**. The outcomes of the multilevel analyses are presented in **Table 2**. Gender was included in all models as a control variable. Contrary to expectations, the intervention had no effect on post-intervention Dutch, English, or mathematics grades: there were no significant main effects of condition and the expected interaction effects between ethnic background and condition were also non-significant. There was a significant effect of gender on Dutch grades: boys performed worse than girls on this subjects. For mathematics, there was a significant effect of ethnic background. As seen in the pre-test, ethnic minority students performed lower on mathematics than ethnic majority students after the interventions.

**Table 1:** Descriptives of grades, problem behavior, belongingness, and identification with school for the different groups in Study 1.

	Native Dutch				Ethnic Minority			
	Control		Affirmation		Control		Affirmation	
	*M*	*SD*	*M*	*SD*	*M*	*SD*	*M*	*SD*
Dutch post-interventions	7.09	0.93	6.94	0.95	6.76	0.83	6.68	0.78
English post-interventions	6.90	1.29	7.00	1.22	6.95	1.28	6.96	1.05
Mathematics post-interventions	7.07	1.10	6.93	1.16	6.63	1.01	6.55	1.12
Problem behavior								
Pre-interventions	1.24	0.25	1.18	0.21	1.23	0.23	1.26	0.30
Post-interventions	1.26	0.31	1.31	0.30	1.28	0.27	1.28	0.30
School enjoyment								
Pre-interventions	2.59	0.34	2.64	0.33	2.54	0.36	2.48	0.39
Post-interventions	2.36	0.46	2.25	0.44	2.28	0.43	2.25	0.48
Perceived social acceptance								
Pre-interventions	2.81	0.33	2.84	0.28	2.78	0.32	2.76	0.29
Post-interventions	2.70	0.44	2.71	0.39	2.74	0.34	2.73	0.34
Identification with school								
Pre-interventions	2.72	0.29	2.80	0.28	2.88	0.24	2.87	0.32
Post-interventions	2.69	0.33	2.59	0.50	2.81	0.33	2.86	0.27

**Table 2:** Effect sizes and standard errors of multilevel models for post-intervention Dutch, English, and mathematics grades Study 1.

	Grades		
	Dutch (*SE*)	English (*SE*)	Mathematics (*SE*)
Gender^a^	-0.53 (0.15)**	-0.13 (0.15)	-0.03 (0.14)
Ethnic Background^b^	-0.41 (0.23)	-0.03 (0.24)	-0.53 (0.23)*
Condition^c^	0.13 (0.14)	0.12 (0.14)	0.06 (0.13)
Ethnic background ^∗^ Gender	-0.46 (0.27)	-0.22 (0.28)	-0.40 (0.26)
Gender ^∗^ Condition	-0.05 (0.21)	-0.32 (0.21)	-0.04 (0.19)
Ethnic background ^∗^ Condition	0.05 (0.26)	-0.11 (0.27)	0.06 (0.25)

*^∗^*p* < 0.05, ^∗∗^*p* < 0.01, ^∗∗∗^*p* < 0.001. Parameter estimates (while controlling for effects of other parameters) can be interpreted as Cohen’s *d*; ^a^Girl = 0, Boy = 1; ^b^Native Dutch = 0, Ethnic minority = 1; ^c^Values-affirmation = 0, Control = 1.*

Because the Moroccan-Dutch and the Turkish-Dutch groups are the largest ethnic minority groups in Dutch society, and because especially Moroccan-Dutch youth is very negatively stereotyped in the Netherlands, analyses were also conducted for these two groups separately, compared to the native Dutch group. The results of these analyses did not differ from those for the ethnic minority group as a whole.

Because we thought it possible that all participants (i.e., native and ethnic minority) could experience some form of stereotype threat, for example because of their low SES (e.g., Bowen et al., 2013), we also examined intervention effects on the group as a whole. Results of these analyses also did not reveal any intervention effects.

As mentioned before, we also conducted analyses in which we controlled for pre-intervention grades. The only difference of these results with the previous models was that pre-intervention achievement was a significant predictor of Dutch, English, and mathematics grades (β = 0.51, *p* < 0.001; β = 0.77, *p* < 0.001; and β = 0.66, *p* < 0.001, respectively). Gender was no longer a significant predictor of post-intervention grades after adding pre-intervention grades to the models.

##### Problem Behavior

Descriptives of pre- and post-intervention problem behavior are displayed in **Table 1**. Three extreme outliers (i.e., deviating more than 4 standard deviations from the mean) were left out of the analyses. In **Table 3** the outcome of the multilevel model is presented. We controlled for pre-intervention levels of problem behavior. Contrary to expectations, the intervention had no significant effect on the problem behavior of ethnic minority students. There was no significant main effect of condition and the expected interaction between ethnic background and condition was also non-significant. Pre-intervention problem behavior was the only significant predictor of problem behavior in this model. The positive parameter estimate of pre-intervention problem behavior indicates that higher levels of pre-intervention problem behavior correspond to higher levels of post-intervention problem behavior. Analyses were also conducted for the Turkish-Dutch and the Moroccan-Dutch group separately. In these models, again only pre-intervention problem behavior was a significant predictor of post-intervention problem behavior.

**Table 3:** Effect sizes and standard errors of multilevel models for post-intervention problem behavior Study 1.

	Problem behavior (*SE*)
Gender^a^	0.09 (0.12)
Ethnic Background^b^	-0.24 (0.17)
Pre-intervention problem behavior	0.57 (0.05)***
Condition^c^	0.07 (0.11)
Ethnic Background ^∗^ Gender	-0.03 (0.22)
Gender ^∗^ Condition	0.05 (0.17)
Ethnic background ^∗^ Condition	-0.30 (0.21)

*^∗^*p* < 0.05, ^∗∗^*p* < 0.01, ^∗∗∗^*p* < 0.001. Parameter estimates (while controlling for effects of other parameters) can be interpreted as Cohen’s *d*; ^a^Girl = 0, Boy = 1; ^b^Native Dutch = 0, Ethnic minority = 1; ^c^Values-affirmation = 0, Control = 1.*

##### School belongingness and identification

Descriptives of school belongingness and identification are displayed in **Table 1**. In **Table 4**, outcomes of the multilevel models are presented. Results show that the only significant predictor of post-intervention school belongingness (i.e., school enjoyment and perceived social acceptance) is pre-intervention belongingness. The positive parameter estimates indicate that the higher the level of pre-intervention belongingness, the higher the level of post-intervention belongingness. For post-intervention identification, ethnic background and pre-intervention identification are significant predictors. Higher pre-intervention identification corresponds to higher post-intervention identification. Moreover, compared to native Dutch students, ethnic minority students report a higher level of identification with school. There was, however, no significant interaction between ethnic background and condition on school identification, which implies that this difference in identification is not related to the intervention.

**Table 4:** Effect sizes and standard errors of multilevel models for post-intervention school belongingness and identification in Study 1.

	Belongingness		
	School enjoyment	Perceived social acceptance	Identification
Gender^a^	-0.03 (0.15)	-0.10 (0.16)	0.02 (0.16)
Ethnic Background^b^	0.11 (0.21)	0.27 (0.22)	0.63 (0.23)**
Pre-intervention belongingness or identification	0.56 (0.05)***	0.48 (0.06)***	0.33 (0.06)***
Condition^c^	-0.05 (0.14)	0.07 (0.15)	-0.25 (0.15)
Ethnic background ^∗^ Gender	-0.34 (0.25)	0.22 (0.27)	-0.17 (0.28)
Gender ^∗^ Condition	-0.06 (0.20)	-0.22 (0.21)	0.20 (0.22)
Ethnic background ^∗^ Condition	0.34 (0.25)	0.04 (0.26)	0.54 (0.28)

*^∗^*p* < 0.05, ^∗∗^*p* < 0.01, ^∗∗∗^*p* < 0.001. Parameter estimates (while controlling for effects of other parameters) can be interpreted as Cohen’s *d*; ^a^Girl = 0, Boy = 1; ^b^Native Dutch = 0, Ethnic minority = 1; ^c^Values-affirmation = 0, Control = 1.*

##### Gender and mathematics performance

The mean post-intervention grade of girls in the affirmation condition was 6.67 (*SD* = 0.79), and of girls in the control condition it was 6.79 (*SD* = 1.06). For boys, the mean post-intervention mathematics grade was 6.54 (*SD* = 0.91) in the affirmation condition and 6.62 (*SD* = 1.01) in the control condition. As can be observed in **Table 2**, there was no effect of gender on post-intervention mathematics performance and the expected interaction between gender and condition was also not significant. When ethnic minority status was removed from the analyses, there was still no significant effect of gender (β = -0.13, *p* = 0.45) and no significant interaction between gender and condition (β = 0.04, *p* = 0.87).

### Discussion

In contrast to earlier findings (Cohen et al., 2006b, 2009), our study did not show that a values-affirmation intervention has any effect on the school performance of ethnic minority students in the Netherlands. Furthermore, no intervention effects were found on school belongingness and identification, which have been assumed to be underlying factors in the process of stereotype threat in US studies (Cook et al., 2012; Sherman et al., 2013). In addition, we also examined whether the values-affirmation intervention would attenuate the level of problem behavior of ethnic minority students, who are often also negatively stereotyped in the behavioral domain. The values-affirmation intervention did not affect the level of problem behavior of these students either. These results are surprising, since intervention procedures were reproduced as closely as possible and ethnic minority students in the Netherlands were assumed to be negatively stereotyped in Dutch society in a manner comparable to African American or Latino American students in the US, and as immigrant students in other European countries, for whom stereotype threat effects have also been demonstrated (Appel et al., 2015). Furthermore, the intervention also did not have an effect on the mathematics performance of girls, who are often negatively stereotyped in the domain of science and mathematics.

Our results may suggest that the intervention simply does not work outside the US. Still, in the US, the intervention had been shown to work for many different negatively stereotyped groups from many different cultural backgrounds. Therefore, we expected that the results would generalize to negatively stereotyped groups outside the US. Furthermore, the previously described study among French nursing students Taillandier-Schmitt et al. (2012) showed that the intervention can have some effect on the performance of negatively stereotyped students outside the US. Also, a study by Thomaes et al. (2012) showed that a values-affirmation intervention can have an effect on the behavior of students in the Netherlands. Although, Thomaes et al. (2012) did not focus on ethnic minority students under stereotype threat, it did show that students who were qualified as antisocial showed elevated levels of prosocial behavior after having completed a self-affirmation exercise comparable to those used in the studies of (Cohen et al., 2006b, 2009).

For these reasons, we decided to test the intervention again, with a different group of students. A difference between previous studies and our first study was that our study took place at the lowest level of secondary education (seventh grade), whereas previous studies (described in the introduction) took place at schools where the performance levels of the students were more mixed. Therefore, we chose to conduct our second study in sixth grade, with students having different performance levels still in the same class. In addition, in Study 2, results of the Cito standardized nationwide test were used as a performance measure, to have a more objective measure of achievement. Furthermore, apart from using only self-reports of problem behavior, which may be subject to social desirability, teachers were asked to report on the problem behavior of a random selection of their students.

## Study 2

Theoretically, it is believed that a values-affirmation intervention works because students are stimulated to reflect on values that are personally important to them. However, self-reflection is often more difficult for younger students. Since the students in our second study were relatively young, we decided to add a third condition to our intervention paradigm. In this condition, the students have a conversation with a teaching assistant after they finish their values-affirmation assignment. This assistant helps them to elaborate on their personally important values. To ensure that effects would still be attributable to the intervention and not simply to the extra attention these students receive, students in the original two conditions (i.e., the values-affirmation and control condition) also have a conversation with the teaching assistant, only their conversation is about a neutral, unrelated, assignment.

Our hypotheses for Study 2 were that the affirmation intervention would positively affect the school performance and the level of problem behavior of ethnic minority students. Furthermore, we expected that the joint elaboration condition would have a surplus effect.

### Materials and Methods

#### Participants

**FIGURE 3:**
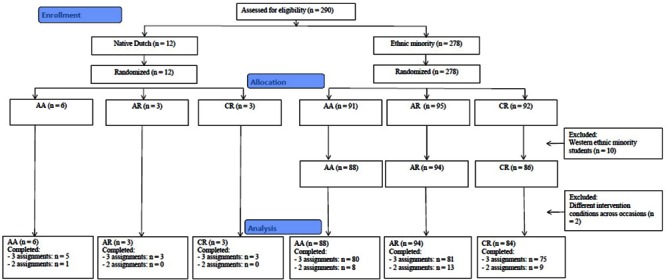
**Flowchart of the random assignment procedure of Study 2**.

In Study 2, 290 students (49.7% boys) participated (12 native Dutch and 278 ethnic minority). Ages ranged from 10 to 13 years (*M*_age_ = 11.28 years, *SD* = 0.57). Condition allocation procedures were the same as in Study 1, except that in the present study there were three conditions, namely an affirmation condition with elaboration on the affirmation assignment (AA), an affirmation condition with elaboration on a non-affirmative unrelated reading task (AR), and a control condition with elaboration on the same unrelated reading task (CR). The assignment procedure is displayed in **Figure 3**.

The study took place in 15 grade six classes at 13 primary schools in the Netherlands. There were two schools with two sixth grades, resulting in 15 participating classes. As our main group of interest were ethnic minority students, schools were again recruited in areas with many ethnic minority students. Percentages of non-Western ethnic minority students ranged between 58 AND 100%.

Of the 278 ethnic minority students, 10 (3.6%) had a Western ethnic minority background. As in Study 1, it was decided to leave these students out of the analyses, leaving 268 non-Western ethnic minority students (88 AA, 94 AR, 86 CR) and 12 native Dutch students (six AA, three AR, three CR) in the analyses. Of these 268 ethnic minority students, 38.8% had a Moroccan-Dutch background, 19.4% had a Turkish background, and 16.4% had a Surinamese or Antillean background. The remaining 25.4% had various backgrounds (e.g., Egypt, Pakistan, Ghana, China, India).

As in Study 1, most ethnic minority students were second generation immigrants (87%), meaning that their parents were born in the country of origin but the children themselves were born in the Netherlands. Thirty-four ethnic minority students (13%) were born in the country of origin of the parents. However, at the onset of the study they had lived in the Netherlands for an average of 7.77 years (*SD* = 3.13).

#### Negative Stereotypes in the Netherlands

To examine if sixth grade ethnic minority students in the Netherlands also experienced more negative stereotyping than native Dutch students, a study was conducted among another sample of fifth and sixth grade students (*N* = 237). Of these students, 47.3% had a native Dutch background, 11.8% had a Moroccan-Dutch background, 8.9% had a Turkish-Dutch background, 11.0% had a Surinamese or Antillean background and 21.1% had another ethnic minority background (eastern Europe, Iraq, Afghanistan, Africa, Asia, South America). Participants were asked to indicate on a three-point scale (yes, often – sometimes – no, never) to what extent they thought that others held the following negative stereotypes about their cultural group: lazy, untrustworthy, aggressive, greedy, dumb, always being late at appointments, done something wrong, criminal, love cheese and wooden shoes, unable to understand things quickly, not hardworking, stealing things, receiving bad grades at school, quickly starts to fight or scold, speaks Dutch badly. Items were rescored, so that a higher score indicated more experience with the stereotype. Multilevel analyses showed that ethnic minority students experienced significantly more often that others saw their cultural group as lazy, untrustworthy, aggressive, dumb, criminal, unable to understand things quickly, not hardworking, stealing things, quickly starts to fight or scold, and speaks Dutch badly. On the other hand, native Dutch students more often experienced that others saw their cultural group as people who all love cheese and wooden shoes. Moreover, when asked on a three-point scale how much they were bothered by the stereotypes that others hold about their group, ethnic minority students indicated to be significantly more often bothered by the negative stereotypes than native Dutch students. These results show that ethnic minority students in fifth and sixth grade thus experience negative stereotyping in the intellectual as well as the behavioral domain, and they feel bothered by these negative stereotypes more than native Dutch students do.

#### Intervention

As in Study 1, the students received three writing assignments throughout the school year. On Occasion 1 they received a writing assignment similar to the one used in Study 1. Because the students in Study 2 were relatively young, they received a simplified intervention on Occasions 2 and 3 (Sherman et al., 2013). On Occasion 2 students in the affirmation conditions again received the list of values and were asked to indicate only their most important value and to write about how that value would be important to them in the coming two winter months. For simplification, the reinforcing questions from Study 1 were removed. In the control condition, the students were asked to give a description of how they usually spend their afternoon, after school is out (e.g., How do you get home? How long does it take you to get home? Do you have a snack? At what time do you go to sleep?). On the third occasion students in the affirmation condition received a tailored writing assignment that stated which value they had chosen on Occasion 2, and asked them to write about why that value would again be important to them in the coming months. Because of an administrative error, there was one class for which the research team did not receive the writing assignments of Occasion 2 in time to prepare the tailored assignments. For these students a tailored assignment was made on the basis of the most important value from Occasion 1. In the control condition students were reminded that on the previous occasion they were asked to write something about how they usually spend their afternoon after they get home from school. Now, they were asked to write about what they had done in the morning, before they had come to school (e.g., At what time did you get up? How long did it take you to get ready? What, if anything, did you have for breakfast? How did you get to school?).

The writing assignments took approximately 15 min to complete. On Occasion 1, the students received an additional reading comprehension test after their writing assignment. They were asked to read a text about ice-cream and answer six questions about this text afterward. The two assignments together took approximately 25 min to complete.

On Occasion 1 all the students had a 10-min conversation with a trained member of our research team about one of their exercises. For participants to believe that the assignments were school exercises, the member of the research team presented herself as a trainee teacher. After the students had finished their assignments, the teaching assistant took the students out of the classroom one by one. In the AA condition, the teaching assistant spoke with the student about the values-affirmation writing assignment. In their writing assignment, students had chosen the two or three values that were most important to them. During the conversation, the students were asked to choose the most important of those two or three values and were asked, with standard questions, to elaborate on why that value was important to them. If there was still time left after discussing the most important value, the second most important value was also discussed. The teaching assistant helped the participant with self-reflection by paraphrasing and summarizing what the participant had said. In the AR and the CR conditions, the conversation was about the reading comprehension assignment. The teaching assistant reflected with the student on how they solved the questions that were part of the assignment and how they solve questions of reading comprehension assignments in general. In this condition, the researcher also paraphrased and summarized the answers of the student. Importantly, in none of the conditions did the teaching assistant give any evaluation of the performance of the student. The researcher made sure each conversation lasted for 10 min.

As in Study 1, Occasion 1 was timed as early in the school year as possible. On Occasions 2 and 3 the writing assignment was presented by the class teacher. Teachers were instructed to schedule the writing assignments directly before a school test. As in Study 1, teachers and students were blind to the purpose of the writing assignments and were unaware of the existence of different conditions.

#### Procedure

**FIGURE 4:**
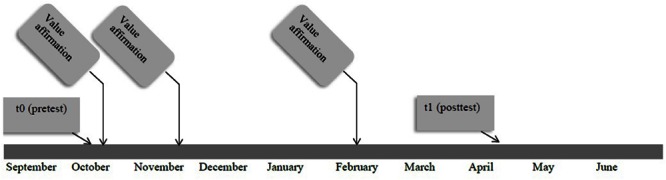
**Time schedule of the intervention Study 2**.

The procedure was similar to that of Study 1 and the time schedule is displayed in **Figure 4**. Five students did not receive parental permission. They received the control writing exercise and an alternative assignment during the measurement occasions.

#### Measures

##### School performance

Results of the Cito standardized test from the end of sixth grade were obtained from the school at the end of the school year. Some students also receive a standardized Cito test at the end of fifth grade, as a pre-indicator of their score on the test at the end of sixth grade. When possible, the schools also supplied the results of this test, to serve as a baseline performance indicator.

##### Problem behavior

Self-reported problem behavior was measured in the same way as in Study 1. Additionally, in this study, teachers also reported on the problem behavior of their students. They were asked to fill in the subscale ‘Conduct problems’ of the Dutch version of the Strengths and Difficulties Questionnaire (SDQ; Van Widenfelt et al., 2003). Because of the large workload of the teachers, we asked them to fill in these questionnaires for 12 randomly selected students from their class. Both student- and teacher-reported problem behavior were measured pre- and post-intervention. The teacher-reported problem behavior of the ethnic minority students in our study was compared to norm scores of the SDQ from 2013, which had been collected among a national sample of 2136 students in regular education, between the ages of 9 and 13 years (Diepenmaat et al., 2014). The percentages of ethnic minority students in the schools that participated in this national study were slightly higher than in the total population. The results showed that compared to the average of this norm population, the teachers in our study reported significantly higher levels of problem behavior for the ethnic minority students in our sample, *t*(117) = 0.57, *p* < 0.01. Since the native Dutch group in our sample was very small (*N* = 12), they were not compared to the norm group.

#### Statistical Analyses

Analytic procedures similar to those used in Study 1 were applied, with two differences. First, in the present study native Dutch students were left out of the analyses. This was done because the group of native Dutch students was very small (*N* = 12) and our main interest was to examine intervention effects for ethnic minority students. Secondly, since there were now three conditions, two dummy variables were created for condition instead of one.

### Results

#### Data Preparation

Due to administrative errors, two participants did not receive the same intervention condition on all three occasions and were excluded from the analyses, leaving 266 ethnic minority students in the analyses (see **Figure 3**). Data are included in the Supplementary Materials (see Data Sheet 2). The majority of the students completed all three writing assignments. Due to administrative errors at one school, one class (*N* = 15) only completed the first and the third assignment. Most of those students who were absent during one occasion completed the assignment once they returned at school. Some students did not receive this opportunity from their teacher (*N* = 16) and completed only two out of three assignments. Only one student missed the first assignment. There were no differences between the three conditions in the mean number of interventions completed, *F*(2,275) = 0.39, *p* = 0.68. Therefore, all 266 ethnic minority students remained in the analyses. The distribution of the students across the three conditions is displayed in **Figure 3**.

For all classes, data on post-intervention school performance (i.e., Cito-scores) were available. For five of the 15 classes (79 ethnic minority students), pre-intervention performance information was available (i.e., the scores on the standardized Cito test from the end of fifth grade). Just as in Study 1, we expected no differences between the students for whom pre-intervention performance was available and those for whom it was unavailable, because the data was missing per class and not per individual student. Indeed, there were no age differences between the five classes for which the data on pre-intervention performance were available and the 10 classes for which these data were not available, *t*(272) = -1.66, *p* = 0.10.

Data on self-reported problem behavior were available for all classes. Three teachers were unable to report on problem behavior at either the first occasion or the second occasion. One class switched teachers three times during the school year. The 11 remaining teachers completed the questionnaires both on Occasions 1 and 2, resulting in teacher reports of problem behavior for 115 ethnic minority students (38 AF, 40 AN, 37 C).

#### Intervention Effects

##### School performance

Descriptives of pre- and post-intervention Cito-scores are displayed in **Table 5**. The outcomes of the multilevel analyses are displayed in **Table 6**. Gender was added as a control variable. Contrary to expectations, the intervention again had no effect on school performance. All other effects were also non-significant.

**Table 5:** Descriptives of grades, problem behavior, belongingness, and identification with school for the different groups in Study 2.

	Native Dutch						Ethnic minority					
	Affirmation + Feedback		Affirmation + Neutral		Control		Affirmation + Feedback		Affirmation + Neutral		Control	
	*M*	*SD*	*M*	*SD*	*M*	*SD*	*M*	*SD*	*M*	*SD*	*M*	*SD*
Cito scores Grade 6	530.50	11.36	527.33	5.69	531.33	8.08	530.43	11.24	529.80	10.26	530.21	11.71
**Problem behavior**									
SR pre-interventions	1.20	0.12	1.52	0.34	1.55	0.36	1.20	0.23	1.25	0.30	1.29	0.30
SR post-interventions	1.16	0.14	1.36	0.48	1.33	0.42	1.20	0.25	1.21	0.27	1.21	0.24
TR pre-interventions	1.70	0.81	1.53	0.50	1.55	0.35	1.28	0.45	1.26	0.34	1.33	0.44
TR post-interventions	1.50	0.50	1.20	0.00	1.60	0.57	1.29	0.44	1.30	0.39	1.30	0.43

*SR, self-report; TR, teacher-report.*

**Table 6:** Effect sizes and standard errors of multilevel models for post-intervention Cito scores of sixth grade Study 2.

	Cito score (*SE*)
Gender^a^	0.25 (0.20)
Dummy 1: AA vs. CR^b^	0.17 (0.20)
Dummy 2: AR vs. CR^c^	0.15 (0.20)
Gender ^∗^ Dummy 1	-0.15 (0.28)
Gender ^∗^ Dummy 2	-0.28 (0.28)

*^∗^*p* < 0.05, ^∗∗^*p* < 0.01, ^∗∗∗^*p* < 0.001. Parameter estimates (while controlling for effects of other parameters) can be interpreted as Cohen’s *d*; ^a^Girl = 0, Boy = 1; ^b^CR = 0, AR = 0, AA = 1; ^c^CR = 0, AA = 0, AR = 1.*

As in Study 1, we also examined intervention effects for the Moroccan-Dutch group and the Turkish-Dutch group separately. The effect was not significant for any of these groups. As in Study 1, we also examined intervention effects for the total group, including the native Dutch students. Again, there were no significant effects.

We also conducted analyses in which we controlled for pre-intervention performance. As mentioned before, these were available for five classes. The only difference was that pre-intervention performance was a significant predictor of post-intervention performance (β = 0.89, *p* < 0.001).

##### Problem behavior

Descriptives of pre- and post-intervention problem behavior are displayed in **Table 5**. The results of the multilevel analyses are displayed in **Table 7**. Gender and pre-intervention student-reported problem behavior were added as control variables. Again, the intervention did not have the expected effect on student-reported problem behavior. The only significant predictor of post-intervention problem behavior was pre-intervention problem behavior. The positive parameter indicates that the higher the level of pre-intervention problem behavior, the higher the level of post-intervention problem behavior.

**Table 7:** Effect sizes and standard errors of multilevel models for post-intervention problem behavior Study 2.

	Self-reported problem behavior (*SE*)	Teacher-reported problem behavior (*SE*)
Gender^a^	0.12 (0.17)	0.13 (0.21)
Pre-intervention problem behavior	0.59 (0.05)***	0.78 (0.06)***
Dummy 1: AA vs. CR^b^	0.15 (0.17)	-0.07 (0.21)
Dummy 2: AR vs. CR^c^	0.03 (0.17)	-0.06 (0.21)
Gender ^∗^ Dummy 1	-0.23 (0.24)	-0.06 (0.29)
Gender ^∗^ Dummy 2	0.08 (0.23)	-0.24 (0.29)

*^∗^*p* < 0.05, ^∗∗^*p* < 0.01, ^∗∗∗^*p* < 0.001. Parameter estimates (while controlling for effects of other parameters) can be interpreted as Cohen’s *d*; ^a^Girl = 0, Boy = 1; ^b^CR = 0, AR = 0, AA = 1; ^c^CR = 0, AA = 0, AR = 1.*

Again, analyses were also conducted for the Moroccan-Dutch group and the Turkish-Dutch group, separately. As before, the only significant predictor in both groups was pre-intervention problem behavior. Analyses were also conducted for the native Dutch group and the ethnic minority group combined. Again, the only significant predictor was pre-intervention problem behavior.

As to teacher-reported problem behavior, there was also only a significant effect of pre-intervention teacher-reported problem behavior. Analyses were also performed for the Moroccan-Dutch and the Turkish-Dutch groups separately. The only difference from the results of the total ethnic minority group was that there was a significant main effect of gender for the Turkish-Dutch group, indicating that teachers reported higher levels of problem behavior for Turkish boys than for Turkish girls at the end of the school year. The analysis with the native Dutch and ethnic minority group combined also yielded the same results as the main analysis.

## General Discussion

The present study investigated whether the positive effects of a values-affirmation on the school performance of ethnic minority students found in earlier studies (e.g., Cohen et al., 2006b, 2009), would generalize to a negatively stereotyped minority group (i.e., immigrant students in the Netherlands) outside the US. With two double-blind field experiments, we examined if the intervention would reduce the negative effects of stereotype threat on the school achievement of ethnic minority students in the Netherlands. In contrast to findings of the previous studies discussed in the introduction, the values-affirmation intervention did not affect the school performance of the ethnic minority students included in our studies. In our second study, a third condition was added to the original paradigm, in which students received help in reflecting on their personally important values. This additional help with self-reflection did not improve their performance either. In addition, we applied stereotype threat theory to the problem behavior of ethnic minority students. We examined if the values-affirmation intervention would attenuate the level of problem behavior of ethnic minority students, who are often also negatively stereotyped in the behavioral domain. However, the values-affirmation intervention did not affect student problem behavior either. Finally, we also did not find that the intervention had an effect on school belongingness and identification, which have been identified as underlying factors in the process of stereotype threat in US studies (Cook et al., 2012; Sherman et al., 2013). These results were unexpected, since the intervention procedures were closely reproduced.

A possible explanation for the absence of intervention effects could be that there are certain preconditions that need to be fulfilled before the intervention can have the intended effect. Perhaps these preconditions were not met in the present study. Although not always explicitly mentioned, several possible preconditions can be deduced from previous studies. For example, a salient achievement gap seems to be necessary for the intervention to work. In most previous intervention studies, there was an achievement gap within the classrooms between the stereotyped and the non-stereotyped group, which was attenuated by the values-affirmation intervention (e.g., Cohen et al., 2006b; Sherman et al., 2013; Harackiewicz et al., 2014). In contrast, in our studies, one could argue that there was no salient achievement gap. In Study 1, there was no pre- or post-intervention gap in English or Dutch. In Study 2, the native Dutch group was very small (*N* = 12) and therefore, ethnic minority students probably did not perceive a gap between their performance and that of the native Dutch students, at least not within the classroom. Could it be that the absence of a clear gap is the reason that the intervention did not have the intended effect in the present study?

An argument against this is that in Study 1, we did find an achievement gap in mathematics. If a salient achievement gap is necessary, then we would have expected at least an intervention effect on mathematics. However, this was not what we found. Furthermore, we believe that although achievement gaps might not have been salient within the classroom, the students most likely did perceive gaps between themselves and other students in broader society. For example, the students in Study 1 had just made the transition from primary to secondary school and were sent to the lowest level of secondary education. Because of this transition, the students were probably very aware of the fact that there are students with higher educational levels. Furthermore, the students in both the first and the second study scored significantly below the national average on nationwide standardized tests that are used to determine to which school level the student should go after sixth grade. Students spend a great amount of time training for these tests and they are most probably very well aware of their own score as well as of the national average. To conclude, we assume that the students in our sample were very aware of existing achievement gaps between themselves and other students. If perceiving a gap is a precondition for the efficacy of the intervention, we believe that this condition was satisfied in our studies.

On the other hand, the precondition of a salient achievement gap may not have been met for girls in mathematics. Within the classes, there was no pre-intervention achievement gap between girls and boys on mathematics performance. Furthermore, previous studies have shown that differences between girls and boys on mathematics performance have dramatically diminished in the past decades (e.g., Else-Quest et al., 2010). Whereas for ethnic minority students there are still plenty of clues that point to achievement gaps in broader society even though there is no gap within the classroom, for girls in mathematics even a gap in society is absent. This absence of a gap may have reduced the working of the intervention for girls in mathematics, even though they may still be negatively stereotyped in this domain.

Another possible precondition that can be deduced from previous research is that students should experience some form of stereotype threat or identity threat (e.g., Cohen et al., 2006b; Bowen et al., 2013; Sherman et al., 2013). One could argue that the intervention did not have the intended effect in the present study because the students in our studies perhaps did not experience stereotype threat, or that they experienced it to a lesser extent than for example African Americans or Latino Americans in the US. However, we have ample reason to believe that ethnic minority students in the Netherlands are pervasively negatively stereotyped in the same manner as the ethnic minorities in previous studies from the US. First, they are negatively stereotyped in the intellectual as well as the behavioral domain (e.g., Verkuyten and Thijs, 2000), and stereotype threat effects have been shown in immigrant students with similar ethnic backgrounds in neighboring European countries (i.e., France, Austria and Germany; Chateignier et al., 2009; Berjot et al., 2011; Appel, 2012; see Appel et al., 2015 for an overview). Furthermore, they also often have a low SES, which is also often accompanied by negative stereotypes (e.g., Croizet and Claire, 1998). Still, even though the level of negative stereotyping in the US and the Netherlands seems similar, it could be argued that the students in our studies *experienced* less stereotype threat than students in previous studies (e.g., Cohen et al., 2006b, 2009). One might, for example, expect that students in classes with an even distribution of ethnic minority and majority students experience more stereotype threat, because the out-group, and therefore also the negative stereotypes, are presumably more salient. Cohen et al. (2006b) for example used a school with a relatively even distribution of African Americans and European Americans. In contrast, in 11 of the 17 classes in our first study, the percentage of ethnic minority students was higher than 90%. The same was true for 13 of the 15 classes in Study 2. Perhaps this high percentage of ethnic minority students made these students experience less stereotype threat. If the experience of stereotype threat is indeed a precondition for the efficacy of the intervention, this could explain the absence of intervention effects. Yet, there are several arguments against this. Because in the present study there were more schools and classes included than in the study of (Cohen et al., 2006b, 2009), it was possible to examine intervention effects separately for the six classes with a relatively even distribution of ethnic minority and majority students (i.e., percentages of ethnic minority students ranging between 50 and 71%). There were still no intervention effects found. Furthermore, in the study of Bowen et al. (2013) the ethnic majority group was also very small and yet in their study the intervention did have the intended effect. The percentage of ethnic minority students in the classroom thus does not seem to determine the level of experienced stereotype threat. Rather, the level of negative stereotyping in broader society seems to matter.

In previous research, some moderators were identified that could potentially influence how much stereotype threat individuals experience. Based on the literature, gender-group identification and gender role orientation might moderate stereotype threat effects for women’s mathematic performance (Schmader, 2002; Tempel and Neumann, 2015). With respect to stereotype threat for ethnic minority students, stigma consciousness might be of influence (Brown and Pinel, 2003). Additionally, individual vulnerability for stress responses, such as trait worry (Tempel and Neumann, 2014), has been shown to moderate stereotype threat effects. Although, it is recommendable that future studies include these potential moderators, we do not expect that they diminished the experience of stereotype threat in the present study. First, there was no reason for us to assume that trait worry would be different for ethnic minority students in the Netherlands than for ethnic minority students with the same background in France and Germany, where stereotype threat effects were found among immigrant students (Chateignier et al., 2009; Berjot et al., 2011; Appel, 2012). Second, previous research has shown that ethnic minority students in the Netherlands identify more strongly with their ethnic group than native Dutch students (e.g., Verkuyten and Thijs, 2001). Finally, because of the frequent negative media attention regarding ethnic minorities in the Netherlands, we expect that the students in the present study were highly aware of their stigmatized status.

Previous studies have merely assumed that experiencing stereotype threat is a necessary precondition for the intervention to work. A values-affirmation intervention is aimed at re-affirming self-integrity. Previous studies have shown that only negatively stereotyped students (e.g., African Americans; Cohen et al., 2006b) benefit from the intervention, whereas non-stereotyped individuals (e.g., European Americans) do not. The assumption is that this is because only negatively stereotyped students are in need of re-affirmation of their self-integrity. Although this assumption seems plausible, stereotype threat was never actually measured in any of these previous studies. Moreover, values-affirmation interventions have also been shown to have positive effects without stereotype threat being present (e.g., Thomaes et al., 2012; or after receiving negative feedback: Koole et al., 1999). Therefore, one cannot be certain that the experience of stereotype threat is indeed a precondition for the intervention to work. To our knowledge, no previous studies have found an adequate way to assess stereotype threat. Future studies should focus on this issue.

To conclude, previous research has provided clues as to what preconditions are important for the efficacy of the intervention. Our study seems to meet these criteria. This leads us back to the question as to why no intervention effects were found in the present study, whereas in previous research the intervention was very successful in improving the performance of negatively stereotyped students. We believe that there are also some broader, contextual, factors that could perhaps explain differences in intervention results between previous studies and the present study. One important difference is that in the present study, unlike in previous studies, most ethnic minority students had a Moroccan or Turkish background, which usually implied that they were Muslims. In their values-affirmation exercises, these students often selected ‘religion’ as one of their most important values. In Dutch society, Muslims are increasingly negatively stereotyped because there is growing media attention for extremism and terrorism which are often associated with Islam (e.g., Kamans et al., 2009). Writing about this value could therefore have increased instead of decreased their awareness of and worries about the negative stereotypes that exist about their group. Consequently, the intervention may have reduced instead of increased feelings of self-integrity.

Another important cultural difference is that the African American group differs from the Moroccan-Dutch and Turkish-Dutch group on one of the five cultural dimensions of Hofstede (2001), namely power distance. Power distance can be defined as the degree to which less powerful members of society accept and expect that power is distributed unequally. Cultures with a high power distance are characterized by hierarchical structures, with an emphasis on obedience and respect. The Moroccan and Turkish culture have a relatively high power distance (Hofstede et al., 2002), whereas African American culture has a moderately low power distance (Gibson, 2008), with the emphasis being more on social equality and equal opportunity. Perhaps being a member of a culture with a high power distance makes it difficult for Turkish-Dutch or Moroccan-Dutch students to believe that they can actually change their situation. In our first study for example, students presumably already perceived themselves as being at the lower end of the ‘hierarchy,’ because they are at the lowest level of secondary education. Perhaps these students perceive those who are placed higher in de hierarchy as more capable of inducing change in society than themselves. This view of themselves as not having the power to change their situation could impede the effectiveness of the intervention. Also, differences in power distance between US and Dutch ethnic minority students may have affected the types of values students selected as most important to them during the values-affirmation assignments. Students with a cultural background having a higher power distance (e.g., Moroccan, Turkish) may be inclined to choose values that are seen as important by their community or those higher in the hierarchy, whereas students from a culture with a low power distance (e.g., African American) may choose values that are more personally important to them. Choosing personally important values might be more related to intrinsic motivation, whereas choosing “community values” might be more related to extrinsic motivation. Extrinsic motivation refers to doing something because it leads to a separable outcome. Intrinsic motivation refers to doing things because they are inherently interesting and enjoyable (Ryan and Deci, 2000). As an example, many ethnic minority students in the affirmation condition chose religion as one of their most important values. When inspecting the motivations these students gave for choosing this value, it seemed that they often formulated relatively extrinsic motivations, such as ‘you should do what is asked in Islam,’ or ‘because of my religion, I will go to paradise,’ or ‘everybody in my family is religious.’ We hypothesize that the intervention would perhaps have been more effective if participants had chosen values that were more *personally* important to them, based on intrinsic motivation. More research is needed to explore if and how the choice of values and the motivation behind this influence intervention effects and if differences in power distance influence the efficacy of the intervention.

Apart from examining intervention effects on performance outside the US, the present study was also the first to examine if a values-affirmation intervention affects the level of problem behavior of negatively stereotyped ethnic minority students. However, it did not. The cultural factors described above that may have undermined the effect of the intervention on school achievement also apply to behavior. An additional explanation for the absence of intervention effects on behavior could be the timing of the intervention. In studies aimed at intervening in school results, self-affirmation is assumed to have the strongest effect when it is planned in a context in which the negative stereotype applies (Cohen et al., 2006a, supporting online material), for example just before a school test. To have an effect on problem behavior, however, timing the intervention in a context in which stereotypes about problem behavior are more salient could perhaps result in greater benefits (see for example Thomaes et al., 2012). More research is needed to examine the effects of a properly timed values-affirmation intervention on the problem behavior of negatively stereotyped ethnic minority students. Such research could perhaps best start in the US, where previous intervention effects were found on school achievement.

## Conclusion

Values-affirmation interventions have had strong positive effects on negatively stereotyped ethnic minority students in previous studies (Cohen et al., 2006b, 2009), but the present study shows that these effects do not simply transfer to all negatively stereotyped ethnic minority groups. To cite Yeager and Walton (2011), social-psychological interventions ‘are not magic.’ They can be powerful, but their effects are dependent on the context in which they are used. In our opinion, there is a strong need to examine what the context-dependent conditions are under which values-affirmation interventions either succeed or fail to attain their goal.

## Author Contributions

The research grant was awarded to FCJ, HK, and PJ. All authors made substantial contributions. EJ, FCJ, HK, and PJ worked together on the design of the research project. Data collection, analysis, and writing of the article were carried out by EJ. FCJ, HK, and PJ critically revised the work. All authors gave their approval of the final version of the article.

## Conflict of Interest Statement

The authors declare that the research was conducted in the absence of any commercial or financial relationships that could be construed as a potential conflict of interest.
